# Useful resources: equipment for eye care

**Published:** 2010-09

## Publications

**Figure:**
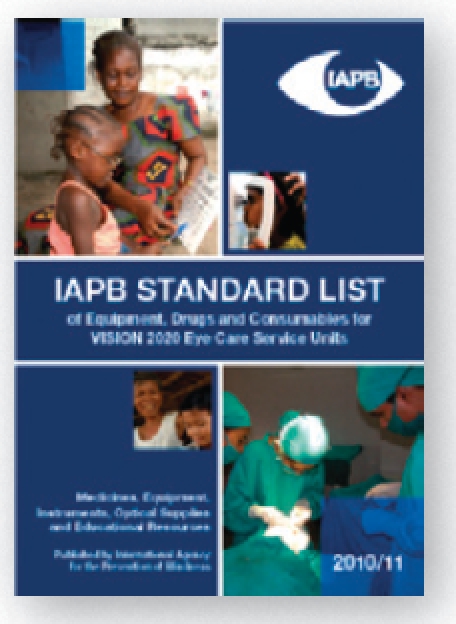


### IAPB Standard List for a VISION 2020 Eye Care Service Unit 2010.

Lists the equipment, instruments, and supplies required by primary and secondary level (district) eye care units, along with suppliers and their addresses. Download from the VISION 2020 website **www.vision2020.org/main.cfm?type=CLINGUIDE** (2.3 MB) or order a copy from TALC (send your name, occupation, and address).

### Stevens S and Naughton A (eds). Technology for VISION 2020.

Contains selected and updated articles on technology for ophthalmic practice from the Community Eye Health Journal. Available for free download from **www.cehjournal.org/files/b0501.html** (1.9 MB, or can be downloaded in smaller sections). Order paper copies from TALC (free to low- and middle-income countries, otherwise £5).

**Figure:**
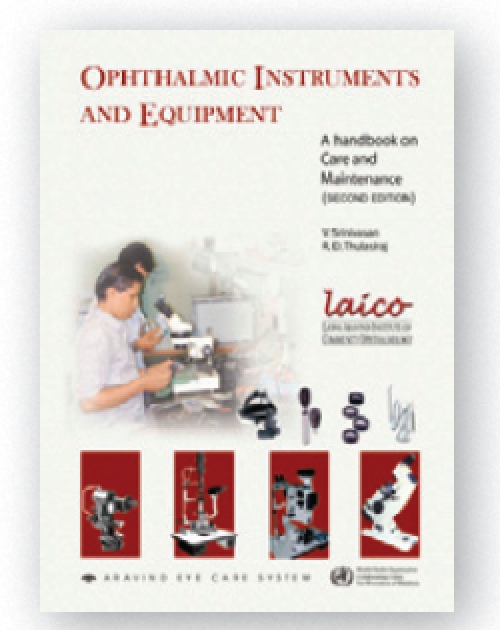


### Srinivasan V and Thulsiraj RD. Ophthalmic instruments and equipment: a handbook on care and maintenance. 2003.

Available from Aravind (UK £1,40/Indian Rs100) Or download from **http://laico.org/v2020resource/files/instruments_book.pdf** (780 kB).

## Video/DVD

### Ophthalmic instruments and equipment: care and maintenance

By V Srinivasan and RD Thulasiraj. A step-by-step guide to the care and maintenance of ophthalmic instruments and equipment.

- Video (PAL format only): order from TALC (UK £7)
- DVD: order from Aravind (UK £7/Indian Rs500). Has English, Spanish, and French language options.

## Past *Community Eye Health Journal* articles

All articles available online at **www.cehjournal.org**

**Vidhya SS, Srinivasan V. Technology for eye care: training in the care of equipment and instruments.** Comm Eye Health J 2002; Issue 43, pages 43-44.

**Srinivasan V. Equipment repaired is equipment gained.** Comm Eye Health J 2009, Issue 70, s02 (online supplement, page 2). **www.cehjournal.org/0953-6833/22/jceh_22_70_s02.html**

**RD Thulasiraj and V Srinivasan. Care of instruments and equipment: a success story.** Comm Eye Health J 2007; Issue 61, page 16.

## Equipment courses

**ORBIS International** conducts two-week workshops on ophthalmic equipment maintenance and health care technology management worldwide. For more information, contact **ismael.cordero@orbis.org** or write to him at ORBIS International, 520 8th Ave, 11th Floor, New York, NY 10018, USA.

**Lions Aravind Institute of Community Ophthalmology (LAICO)** in Madurai runs six-week training courses for technicians that are repeated four times a year (US $325). LAICO offers shorter courses (two, three or four weeks) on invitation at a range of different countries. Visit **www.aravind.org/education/course-details.asp** or write to Prof V Srinivasan, LAICO, 72, Kuruvikaran Salai, Gandhi Nagar, Madurai 625 020, Tamil Nadu, India.

## Other resources

The **Eye Care Equipment Maintainers Discussion Group** is intended as a meeting point for people who use or maintain ophthalmic equipment or are interested in learning more. You can ask questions, share successes, or discuss any problems you are having with your ophthalmic equipment. Visit **http://groups.google.com/group/eye-care-equipment-maintainers to join** in.

Visit the **World Health Organization site http://who.ceb.unicamp.br/** for an up-to-date list of the available training units for biomedical equipment technicians and clinical engineers worldwide. Requires a Flash plug-in.

The **Inter Agency Procurement Group (IAPG)** is a forum for international non-government organisations to share logistical information and procedures; it meets every quarter. Visit **www.iapg.org.uk** for more information.

## Suppliers

**TALC (Teaching Aids at Low Cost):** PO Box 49, St Albans, Hertfordshire, AL 1 5TX, UK. Email: **info@talcuk.org** or visit **www.talcuk.org**

**Aravind:** Mail your order or enquiry to The Manager, Stores, Aravind Eye Hospital, 1, Anna Nagar, Madurai 625 020. Email **stores@aravind.org** or visit **www.aravind.org/publications/catalogue.asp**

